# Effectiveness of a Structured Robot-Assisted Ankle-Foot Sensorimotor Training Program Versus Manual Training in Chronic Stroke: Protocol for an Assessor-Blinded, Parallel-Group Randomized Controlled Trial

**DOI:** 10.2196/104766

**Published:** 2026-09-25

**Authors:** Pınar Kaya, Esra Tekeci, Elif Hocaoğlu, Ramazan Ünal, Gokhan Ozkocak

**Affiliations:** 1 Department of Physiotherapy and Rehabilitation, School of Health Sciences, Istanbul Medipol University, Kavacık, Ekinciler Street, No.19, Istanbul, 34810, Türkiye, +90 216 444 85 44; 2 Department of Physical Therapy and Rehabilitation, Institute of Health Sciences, Istanbul Medipol University, , Istanbul, Turkey; 3 Department of Electrical and Electronic Engineering, Faculty of Engineering and Natural Sciences, Istanbul Medipol University, Istanbul, Türkiye; 4 Department of Mechanical Engineering, Faculty of Engineering, Istanbul Özyeğin University, Istanbul, Türkiye; 5 Department of Physical Medicine and Rehabilitation, Medipol Acıbadem Regional Hospital, Istanbul, Türkiye

**Keywords:** stroke, chronic stroke, robot-assisted rehabilitation, ankle-foot rehabilitation, sensorimotor training, virtual reality, proprioception

## Abstract

**Background:**

Stroke is a leading cause of long-term disability worldwide. Persistent lower-extremity motor and somatosensory impairments after stroke commonly limit walking and balance despite rehabilitation. Virtual reality (VR)–integrated robotic rehabilitation may support structured, goal-directed ankle-foot practice; however, evidence for ankle-foot–focused sensorimotor protocols remains limited. In particular, approaches that combine robot-assisted motor training with plantar tactile localization and VR-supported joint position sense training to target plantar sensory and proprioceptive function are scarce.

**Objective:**

This study aims to evaluate the effectiveness of a structured, VR-integrated, robot-assisted ankle-foot sensorimotor rehabilitation protocol compared with a content-matched manual training protocol in individuals with chronic stroke and to examine its effects on clinical and sensorimotor outcomes.

**Methods:**

This is an assessor-blinded, 2-arm, parallel-group randomized controlled trial. Thirty individuals with chronic stroke will be randomized 1:1 to the robot-assisted training group or the manual training group. All participants will receive conventional rehabilitation. In addition, the robot-assisted training group will receive a structured robot-assisted ankle-foot training program integrated with VR and assist-as-needed control, whereas the manual training group will receive the same structured ankle-foot training protocol delivered manually by a physiotherapist. Interventions will be delivered 3 times per week for 6 weeks, for a total of 18 sessions. Total session duration will be time matched between groups at 50 to 60 minutes per session. The primary outcome will be the change in 10-meter walk test–derived walking speed from baseline to 6 weeks. The secondary outcomes will include 2-minute walk test distance, ankle range of motion, joint position sense, plantar tactile sensation, muscle tone, motor performance, static and dynamic balance, and stroke-specific quality of life.

**Results:**

This study is funded by the Scientific and Technological Research Council of Turkey (TÜBİTAK) under the 1002-A Rapid Support Program (grant 225S390). Recruitment began in March 2026 and is planned to continue until May 2027. As of March 25, 2026, four participants have been enrolled and are currently receiving the assigned intervention. Data analysis will begin once all enrolled participants have completed post-intervention assessments.

**Conclusions:**

This trial will provide evidence on whether a structured robot-assisted ankle-foot sensorimotor training program offers additional benefit compared with a content-matched manual training protocol in individuals with chronic stroke. The findings may contribute to the development of standardized, individualized, and sensorimotor-oriented rehabilitation protocols for improving walking and balance after stroke.

## Introduction

### Background

Stroke is a global public health problem associated with high rates of physical and cognitive disability [1]. Approximately 15 million people experience stroke each year; of these, 5.5 million die, and 5 million are left with lifelong disabilities. Over the last two decades (1990‐2019), the absolute number of stroke events has reportedly increased by 70%, stroke prevalence by 85%, stroke-related deaths by 43%, and disability-adjusted life years by 32% [2].

Although rehabilitation during the first 6 months (the acute phase) can yield substantial neurological and functional recovery, lower-extremity dysfunction and abnormal gait patterns frequently persist even after walking capacity has been regained, limiting activities of daily living [3]. Post-stroke gait impairments are commonly linked to the interaction of spasticity, muscle weakness, and motor control deficits [4]. In particular, ankle-foot spasticity may lead to deformities that compromise knee, hip, and trunk control and contribute to gait anomalies, such as asymmetry, reduced walking speed, and genu recurvatum [5,6]. Post-stroke somatosensory impairments are also common in the lower extremities, and reduced sensory input can negatively affect motor performance [7-10]. The plantar surface and ankle play a key role in balance by providing somatosensory feedback and enabling rapid postural corrections [7,9]. Plantar sensory deficits have been associated with poorer balance, increased postural sway, inadequate weight transfer, higher fall risk, and gait asymmetry, while proprioceptive impairments are linked to difficulties in motor learning and reduced walking speed [7-10]. Abnormal foot postures may alter plantar pressure distribution and load transfer, providing a rationale for interventions targeting plantar pressure patterns and somatosensory feedback [9,11].

Advances in engineering have accelerated the adoption of robot-assisted rehabilitation [12,13]. Compared with conventional therapy, robotic rehabilitation can deliver higher-dose, intensive, and repeatable practice [14]. Sensor-derived physical data enable objective monitoring of training performance, supporting individualized parameter adjustment [14-16]. Integrating virtual reality (VR) into robotic training may further promote active engagement through interactive, function-focused tasks [12,16].

Lower-limb robots are commonly classified as exoskeletons or end effectors [17]. Although exoskeleton-based gait training may improve global walking outcomes after stroke, evidence regarding its effects on balance and general lower-limb motor impairment remains less consistent [17,18]. Consequently, there is growing clinical interest in distal ankle robots as a targeted option to address ankle-specific impairments and tailor training to individual patient needs [19-21]. Distal ankle robots typically train dorsiflexion-plantarflexion and inversion-eversion to improve ankle control and may support maintenance of ankle mobility, with potential benefits for ankle range of motion (ROM) and proprioceptive function [19,20]. These devices can be delivered in modes ranging from passive stretching or ROM to active-assisted or active training [19,20].

Most ankle robotics studies to date have focused on passive ROM and stretching, primarily evaluating feasibility and effects on joint mobility and muscle-related properties across heterogeneous devices and parameter settings [15,21-24]. In parallel, there has been increasing emphasis on multicomponent programs combining passive applications with active or active-assisted training, with reported benefits for active ROM, strength, spasticity, balance, and gait outcomes [16,22,23]. However, protocols remain heterogeneous in duration, speed, ROM, feedback, and control strategies, and evidence from systematic comparisons with manual or conventional approaches is still limited [15,24]. Moreover, relatively few studies have examined structured control strategies that monitor performance in real time and dynamically adjust task difficulty during active ankle ROM training [16,23].

Robot-based approaches can promote active participation through VR tasks and deliver assistance using an assist-as-needed (AAN) paradigm. Compared with passive, repetition-based practice, these strategies may better support motor learning and cortical reorganization and may enhance neuroplasticity through task-oriented repetition [25,26]. On the basis of this rationale, this study proposes a structured robot-assisted ankle-foot training protocol that combines passive ROM or passive stretching with active or active-assisted ROM training within a single program. Active engagement will be supported using VR-based games and visual feedback, and assistance during the active component will be adjusted in real time according to game performance using an AAN strategy. This protocol aims to standardize both passive and active components while addressing the limited evidence for performance-sensitive, adaptive control during active ankle ROM training. Importantly, plantar somatosensory input is integral to post-stroke balance and gait [9,27,28]. Although vibrotactile stimulation is commonly used to enhance plantar sensation, most studies have focused on stand-alone vibration devices that are not integrated into robotic rehabilitation systems [27,28]. Furthermore, tactile perceptual learning paradigms have been associated with somatosensory cortical coactivation and plasticity [29,30]. However, evidence is lacking for plantar vibrotactile localization training integrated into robot-assisted ankle-foot rehabilitation in people with stroke, despite the presence of tactile mislocalization after stroke [31-34]. Therefore, this study aims to address this gap by structuring plantar vibrotactile sensory training within a robot-assisted, performance-sensitive, and real-time adaptive framework.

### Study Objective and Hypothesis

The primary objective is to compare the effect of structured robot-assisted ankle-foot training versus manual training on walking speed (m/s) derived from the 10-meter walk test (10MWT) in individuals with chronic stroke. The secondary objectives are to evaluate between-group differences in ankle ROM, walking capacity, motor performance, joint position sense (JPS), plantar tactile sensation, static and dynamic balance, muscle tone, and stroke-specific quality of life (QoL).

The primary hypothesis is that the robot-assisted program will yield greater improvement in 10MWT-derived walking speed (m/s) than manual training, which includes comparable ankle-foot sensorimotor training components delivered by a physiotherapist. The secondary hypotheses are that the robot-assisted program will yield greater improvements in 2-minute walk test distance; ankle sensorimotor outcomes (ROM, JPS, and plantar tactile sensation); and muscle tone, motor performance, balance measures, and stroke-specific QoL. Finally, we hypothesize that integrating plantar vibrotactile sensory localization and VR-based JPS training within the robotic protocol will be feasible to deliver in this population, as reflected by session completion or adherence and safety monitoring.

## Methods

### Trial Design

This is an assessor-blinded randomized controlled trial with 1:1 allocation comparing structured robot-assisted ankle-foot training (robot-assisted training group [RTG]) with structured manual ankle-foot training delivered by a physiotherapist (manual training group [MTG]) in individuals with chronic stroke. Manual training was chosen as an active comparator because it includes similar therapeutic targets and training components to the robot-assisted program, but without robotic assistance, VR feedback, or automated sensorimotor control. This design enables evaluation of the added value of the robot-assisted intervention over a clinically relevant manual training approach. The findings of this trial will be reported in accordance with the CONSORT (Consolidated Standards of Reporting Trials) guidelines. This protocol is reported in accordance with the SPIRIT (Standard Protocol Items: Recommendations for Interventional Trials) guidelines (Checklist 1).

### Study Setting

The study will be conducted at Acıbadem Medipol University Regional Hospital (Istanbul, Turkey) and the Living Robotics Laboratory (Department of Electrical and Electronics Engineering, Istanbul Medipol University). Eligible participants will be adults with chronic stroke diagnosed by a physician and meeting the study eligibility criteria. To reach the target sample size, outpatient clinic records will be regularly screened, relevant clinicians will be contacted, and assessment and treatment appointments will be planned flexibly whenever possible.

**Figure 1.**
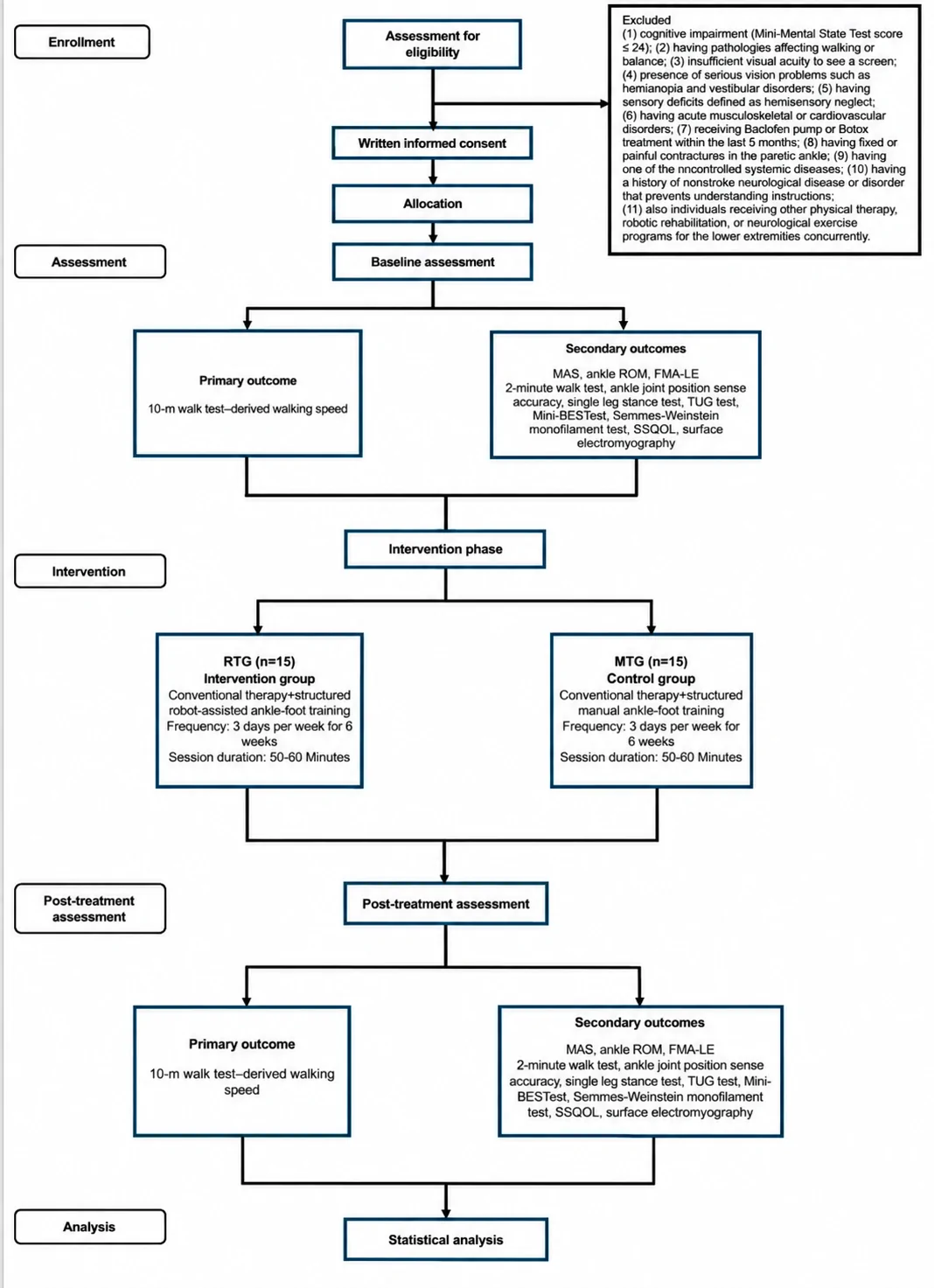
Flow diagram of the trial. FMA-LE: Fugl-Meyer Assessment–Lower Extremity; MAS: Modified Ashworth Scale; Mini-BESTest: Mini Balance Evaluation Systems Test; MTG: manual training group; ROM: range of motion; RTG: robot-assisted training gTraining Group; SSQOL: Stroke-Specific Quality of Life Scale; TUG: Timed Up and Go.

Interventions will be delivered by physiotherapists with at least 2 years of experience in neurological rehabilitation [35]. Technical setup and device-related adjustments will be performed by engineers, while clinical procedures, participant positioning, safety monitoring, and supervision of treatment parameters will be the responsibility of the physiotherapy team. Outcomes will be assessed by a blinded physiotherapist at baseline (T0) and post-intervention (T1). Recruitment began in March 2026 and is expected to continue until May 2027. The study flowchart is presented in Figure 1, and the schedule of enrollment, interventions, and assessments is shown in Table 1.

**Table 1.** Schedule of enrollment, interventions, and assessments.

Study procedures and assessments	Trial period			
	Enrollment		Post-randomization	
Time point	t_i_ to 0	0	T1	T2
Eligibility screening	✓			
Informed consent	✓			
Randomization	✓			
Primary outcome measure				
10-meter walk test–derived walking speed		✓	✓	✓
Secondary outcome measures				
Passive and active ankle range of motion	✓		✓	✓
Fugl-Meyer assessment for the lower extremity	✓		✓	✓
2-minute walk test		✓	✓	✓
Modified Ashworth Scale	✓		✓	✓
Ankle joint position sense		✓	✓	✓
Single-Leg Stance Test		✓	✓	✓
Timed Up and Go Test		✓		
Mini Balance Evaluation Systems Test		✓		
The Semmes-Weinstein Monofilament Test		✓		
The Stroke-Specific Quality of Life Scale		✓		
Interventions				
Robot-assisted training group			●───────────●	
Manual training group			●───────────●	

### Eligibility Criteria

The inclusion criteria are as follows: (1) age 40 to 65 years, (2) able to understand and follow study instructions, (3) able to communicate coherently and oriented to time and place, (4) provided written informed consent, (5) stroke ≥6 months prior to enrollment (chronic stroke), (6) ankle plantarflexor spasticity ≤2 on the Modified Ashworth Scale [15], (7) ankle dorsiflexor strength ≥grade 2 on the Medical Research Council scale [15], (8) passive ankle dorsiflexion to neutral (90°; 0°) without a plantarflexion contracture [15], (9) moderate or mild lower-extremity impairment based on the Fugl-Meyer Assessment–Lower Extremity (FMA-LE) score (21‐27, moderate; and 28‐34, mild or good) [36], (10) able to sit for at least 1 hour [37], (11) able to walk at least 10 m with or without an assistive device [20], and (12) completed all conventional lower-extremity physical therapy and rehabilitation programs.

The exclusion criteria are as follows: (1) cognitive impairment (Mini-Mental State Test score ≤24); (2) conditions affecting walking or balance (eg, orthopedic complications, lower-extremity amputation, and osteoporosis) [22]; (3) insufficient visual acuity to view a screen (eg, diplopia) [22]; (4) severe visual deficits (eg, hemianopia) or vestibular disorders [32]; (5) sensory deficits such as hemisensory neglect; (6) acute musculoskeletal or cardiovascular disorders [23]; (7) intrathecal baclofen pump use or botulinum toxin injections within the past 5 months [23]; (8) fixed or painful contracture of the paretic ankle [12,24]; (9) uncontrolled systemic diseases (eg, diabetes, hypertension, and debilitating or immunosuppressive diseases) [15]; (10) history of a nonstroke neurological disease or disorder that may impair comprehension of instructions; and (11) concurrent participation in other lower-extremity physical therapy, robotic rehabilitation, or neurological exercise programs.

### Sample Size

On the basis of the post-intervention walking speed values reported by Cho et al, mean 1.22 (SD 0.33) m/s in the robot-assisted ankle muscle training group, and mean 0.84 (SD 0.29) m/s in the control group, the estimated effect size was Cohen *d*=1.21. Using G*Power 3.1 (version 3.1.9.7; Heinrich Heine University Düsseldorf) for an independent samples *t* test, with a 2-tailed α level of .05, 95% power, and an allocation ratio of 1:1, the required sample size was calculated as 19 participants per group. Therefore, the total sample size was revised to 38 participants.

### Sequence Generation and Allocation Concealment

Randomization will be implemented using a centralized computer-based system (Study Randomizer; Phase Locked Software) by a research coordinator who is not involved in referral, intervention delivery, or outcome assessment. Stratified permuted block randomization will be used to balance groups according to baseline FMA-LE severity (0‐20, severe; 21‐27, moderate; and 28‐34, mild or good) [36] and affected hemisphere (right or left). Within each stratum, participants will be allocated 1:1 using fixed block sizes (block size=4; final block=2 if required). Following eligibility confirmation and completion of baseline assessments, participant details will be entered into the system, which will automatically generate the allocation. The allocation sequence will be concealed within the centralized system; enrolling and treating personnel will not have access to the randomization lists or assignment order, preventing prediction or manipulation of allocations.

### Blinding

This study will be single blind (assessor blinded). Outcome assessments will be performed by a trained research assistant blinded to group allocation and not involved in referral, randomization, intervention delivery, or trial coordination. Due to the nature of the intervention, participants and the treating physiotherapist cannot be blinded.

### Interventions

#### Overview

All participants will receive conventional rehabilitation for approximately 20 to 25 minutes, 3 days per week, for 6 weeks. The conventional program will include upper extremity mobilizations, trunk and upper extremity flexibility or positioning exercises for spasticity, and trunk and balance training in sitting [24].

In addition to the conventional program, participants will receive structured ankle-foot training. The RTG will undergo structured robot-assisted ankle-foot training, whereas the MTG will receive the same structured ankle-foot training protocol administered manually by a physiotherapist. Ankle-foot training sessions will last approximately 30 to 35 minutes, yielding a total session duration of 50 to 60 minutes for both groups when combined with conventional rehabilitation.

#### Robotic System

The BalanSENS robotic rehabilitation system will be used in the RTG. The system includes a fixed, platform-based end-effector ankle-foot robot featuring a parallel manipulator with a 3-degree-of-freedom rotary-prismatic-spherical configuration [38,39]. The usability of the system has been previously evaluated in healthy individuals [39].

#### Robot Design and Features

**Figure 2.**
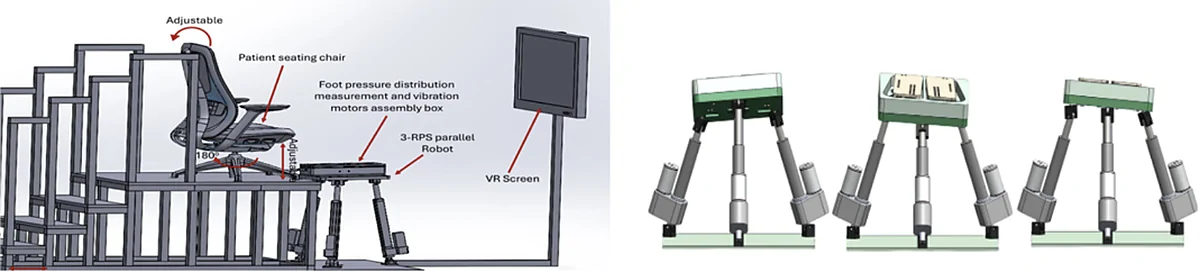
BalanSENS platform and seated foot-ankle rehabilitation setup incorporating a 3-RPS parallel robot, with an illustrative representation of platform motion at different angles. 3-RPS: three-degree-of-freedom rotary-prismatic-spherical; VR: virtual reality.

The system is designed for safe, comfortable seated rehabilitation. The height-adjustable, 180° rotatable chair integrated into the platform facilitates alignment of the participant with the robot. The chair and platform allow adjustment of hip-popliteal length, hip width, shoulder height, and the distance between the participant and the ankle-foot robot (Figure 2). The robot provides 3 degrees of freedom motion (2 rotational axes and 1 linear axis) to train ankle dorsiflexion-plantarflexion and inversion-eversion movements via the 3-degree-of-freedom rotary-prismatic-spherical parallel mechanism, enabling controlled dynamic loading of the ankle [38].

**Figure 3.**
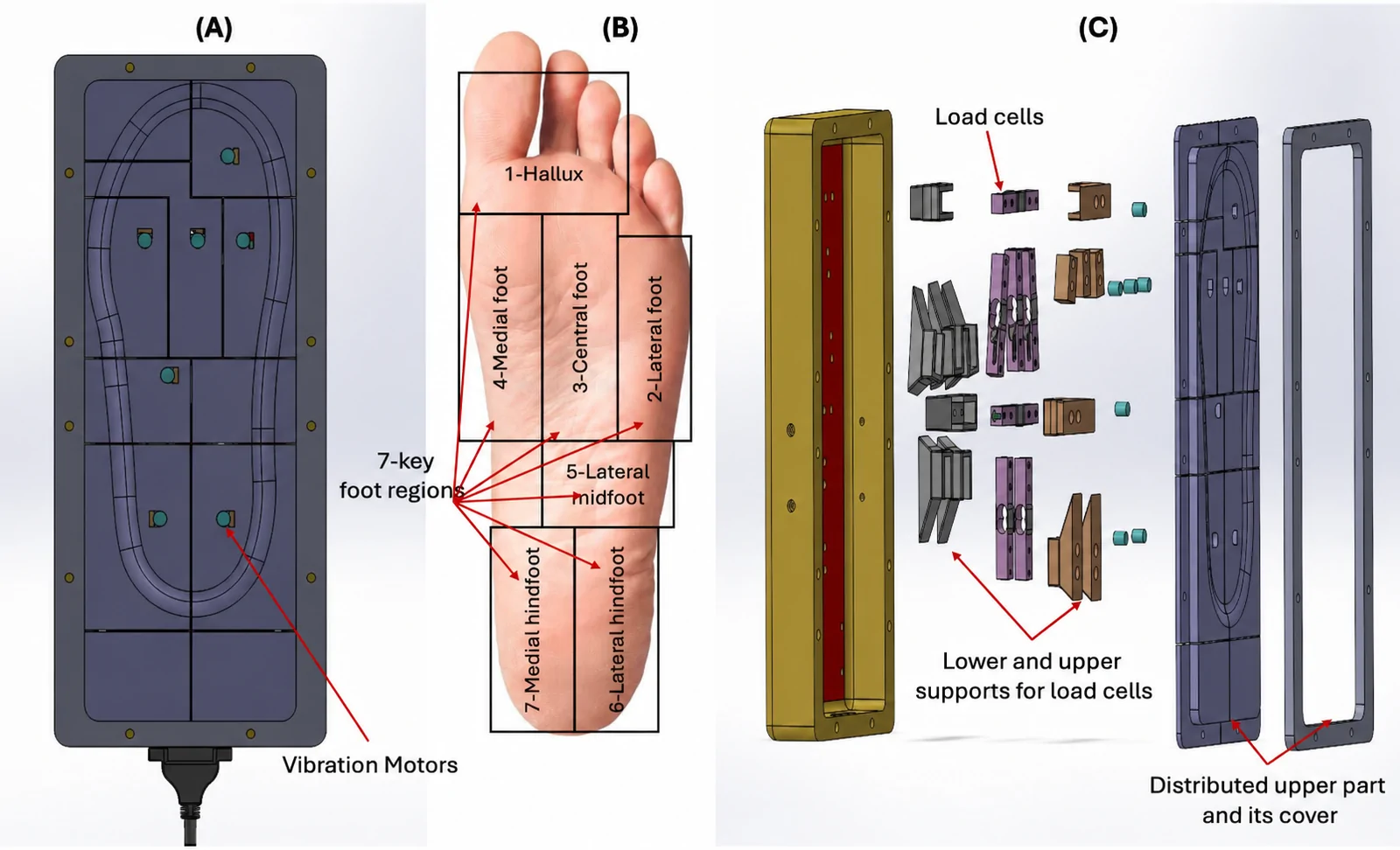
Representation of foot pressure measurement and vibration motor assembly box mounted on the upper platform of the parallel robot: (A) top view of the assembly box, (B) 7 key plantar regions for placement of load cells and vibration motors, and (C) exploded view of the assembly for illustration.

The system includes load cells, vibration motors, and a multiaxis motion platform. Load cells are calibrated using a second-order polynomial for force-voltage conversion, enabling patient-specific torque limits and ensuring a safe ROM [38]. Vibration motors (HUB360) positioned under each plantar region operate at 5 V direct current and provide haptic stimulation (0‐200 Hz frequency; 0%‐100% pulse width modulation duty cycle). A plantar map comprising 7 regions corresponding to motor locations is displayed on the integrated screen, allowing the participant to indicate the perceived stimulation location (Figure 3) [38].

The robot provides position control during passive ROM training and impedance control during active ROM training, allowing both passive and active practice. During passive training, the ankle follows a robot-defined trajectory; during active training, participants perform movement tasks at prescribed speeds with assistive or resistive support guided by visual instructions [40]. The platform-based system operates under an AAN paradigm, monitoring ankle kinematics and kinetics in real time and providing only the assistance required to complete the task. Task difficulty is individualized by adjusting platform stiffness, movement speed, and end-position holding duration (VR tasks).

**Figure 4.**
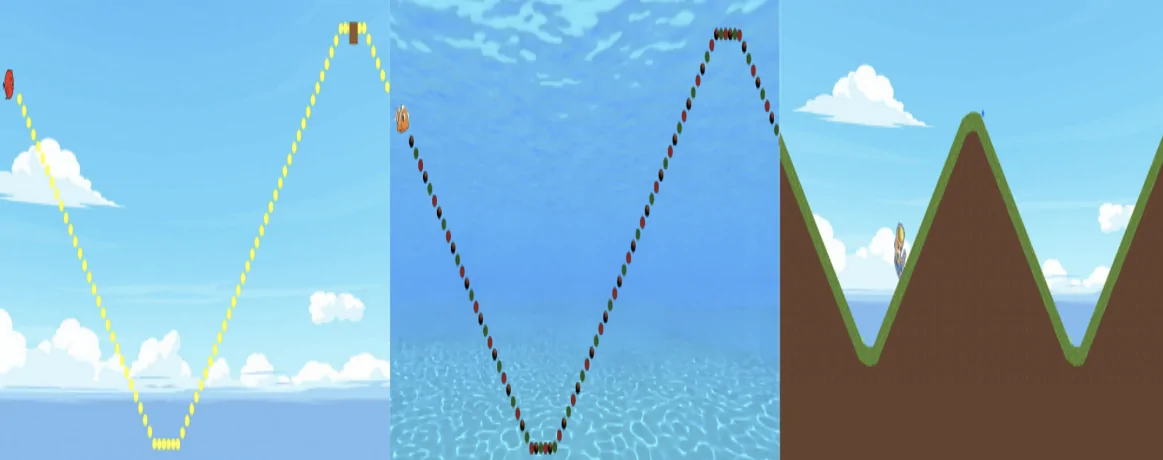
Virtual reality game interface for passive range of motion training, comprising 3 different environments.

**Figure 5.**
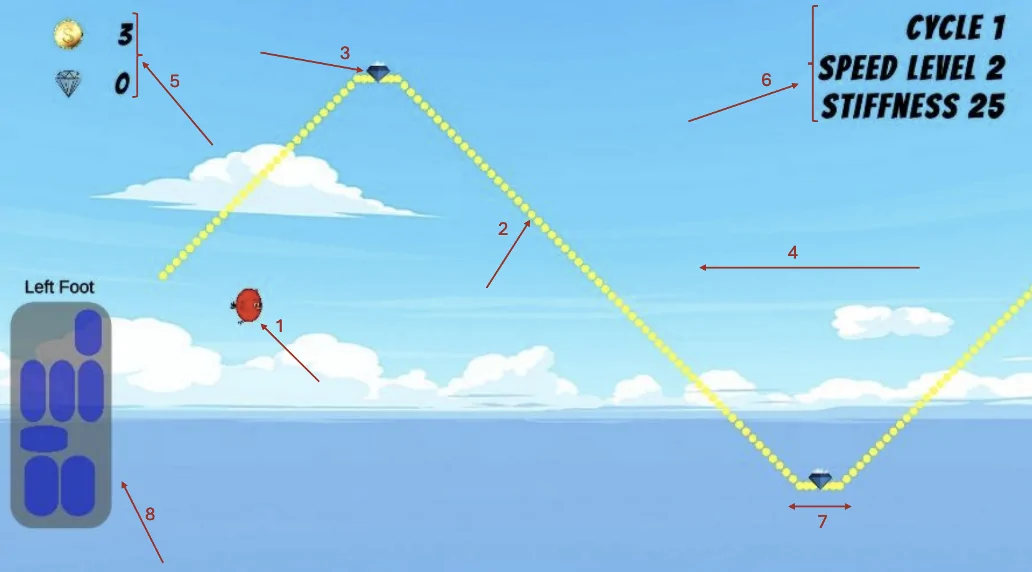
Virtual reality game interface for active training: (1) a bird avatar representing ankle motion along the dorsiflexion–plantarflexion (DP) axis, (2) coins positioned along the movement trajectory to encourage continuous tracking, (3) diamonds placed at the calibrated range of motion (ROM) boundaries to promote full-range excursions, (4) right-to-left trajectory flow, (5) real-time score displaying collected coins and diamonds, (6) therapy parameters shown on-screen (cycle number, speed, and stiffness level), (7) a maximal ROM holding zone providing peak rewards, and (8) real-time plantar pressure distribution derived from footplate sensors.

**Figure 6.**
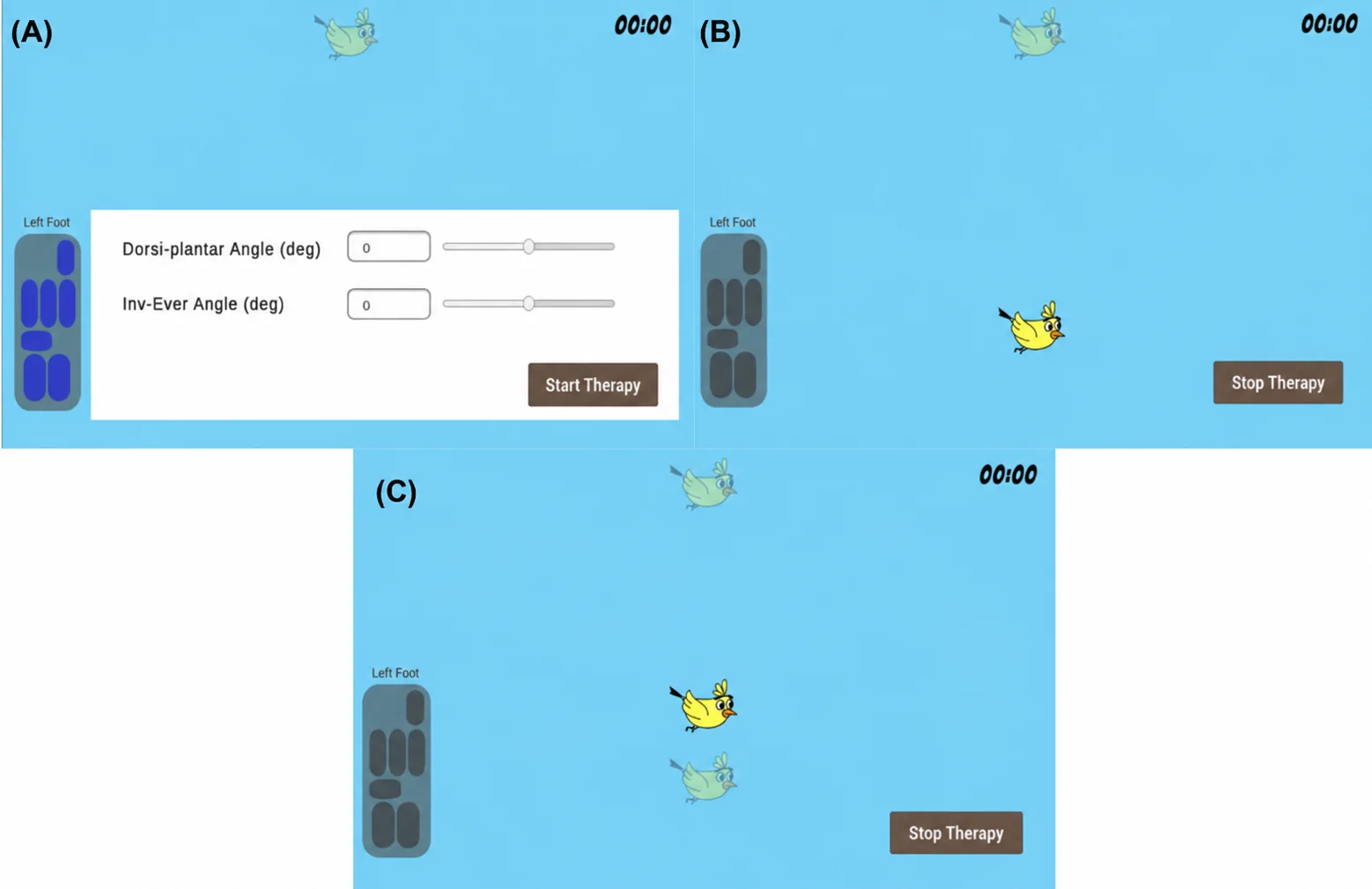
Virtual reality game interface for joint position sense training: (A) the target angle is set and recorded by the system, (B) the robot moves the ankle to the target angle (passive positioning), and (C) the participant is asked to actively reproduce (match) the target angle.

Visual feedback will be provided throughout training using 2 VR-based games. The first VR game will be used during both passive and active ROM training. In this game, ankle dorsiflexion-plantarflexion is represented by the upward-downward motion of a bird to collect targets. During passive training, the bird’s movement follows the robot-driven ankle trajectory (Figure 4), whereas during active training, it is controlled by the participant’s ankle movement (Figure 5). The second VR game is specifically designed for JPS training, in which upward bird movement is linked to dorsiflexion to encourage active participation (Figure 6).

#### Participant Positioning

##### RTG Position

Participants will be comfortably seated on a height-adjustable chair with back support, adjusted so that the hip-knee angle is approximately 90° in accordance with ergonomic criteria. The paretic foot will be placed on a footplate, and the nonparetic foot on a footrest matched to the plate’s height. The paretic foot will be secured using a Velcro strap. A monitor providing interactive VR-based visual feedback will be positioned at the participant’s eye level.

##### MTG Position

Throughout all training stages, participants will be positioned in the long-sitting position with the knee supported at approximately 30° to 45° of semiflexion. This position is intended to ensure balanced muscle-tendon tension and safe implementation of the interventions [41]. As the manual training is delivered without robotic fixation and alignment constraints, long sitting is used to facilitate consistent therapist handling across stages.

### Robot-Assisted Training Protocol

#### Overview

The experimental procedure will consist of a structured, robot-assisted ankle-foot training protocol. The protocol comprises (1) plantar vibrotactile sensory training, (2) passive ROM training, (3) VR-integrated JPS training, (4) active ROM training under an AAN paradigm with real-time adjustment based on VR game performance, and (5) repetition of plantar vibrotactile sensory training.

During robot-assisted training sessions, surface electromyography (sEMG) from the tibialis anterior and gastrocnemius will be monitored in real time on-screen together with load-cell signals to support session supervision and safety (eg, detection of excessive cocontraction or abnormal effort). sEMG monitoring will be used for clinical oversight during training and will not be used as an efficacy outcome.

The training stages and progression are described below.

#### Stage 1: Sensory Education—Plantar Vibration and Sensory Localization Training

The current literature suggests that plantar vibration in people with stroke may positively affect balance, gait, and proprioceptive feedback. However, due to heterogeneity in stimulation parameters, there is no consensus on optimal frequency and amplitude [27]. In people with stroke, plantar interventions have commonly been applied at 80 to 100 Hz [28,42]. In vibrating insoles or wearable systems used during walking, higher frequencies (eg, 200‐250 Hz) have been used to enhance proprioceptive stimulation [42].

In the first part of stage 1, vibration parameters will be individualized based on sensory tolerance. Individual perception and discomfort thresholds will be determined prior to training. The stimulation frequency will initially be set at 80 Hz, and vibration intensity will be gradually increased by adjusting the pulse width modulation duty cycle from 0% to 100%. The clinical stimulation intensity will be selected slightly below the discomfort threshold while still providing a clearly perceived somatosensory stimulus. Following threshold determination, a brief frequency scan (80, 100, 120, and 150 Hz) will be performed at the selected stimulation intensity, and the frequency at which the participant reports perceiving the vibration most clearly will be recorded as the preferred frequency. During training, plantar vibration will be applied within the 80 to 150 Hz range for all participants, with stimulation intensity individualized according to each participant’s sensory tolerance. After determining threshold parameters, all plantar vibration motors will be activated to provide stimulation for 3 minutes, delivered in consecutive cycles of 10 seconds of vibration followed by 5 seconds of rest.

In the second part of stage 1, vibration will be combined with sensory localization training. A 7-zone plantar map corresponding to vibration motor locations will be displayed on the integrated screen (Figure 3). The participant will indicate the perceived stimulation zone using a wireless mouse. Each set will consist of 12 stimulation sequences randomly selected from the 7 plantar zones (eg, 1-7-5-3-1-2-4-6-5-7-3‐4). Each stimulation will be delivered for the planned stimulus duration (Table 2), followed by a fixed 5-second rest or response period with no additional stimulation, during which the participant identifies the perceived zone. Correct responses and reaction times will be recorded as monitoring indicators to track sensory-performance progression throughout training (not as outcome measures). Progression at this stage will be achieved by gradually reducing stimulus duration and increasing the number of sets (Table 2).

**Table 2.** Progression of training stages for both groups.

Stage and parameter	Weeks 1 and 2	Weeks 3 and 4	Weeks 5 and 6
Sensory localization training			
Stimulation duration	5 seconds	4 seconds	3 seconds
Number of sets	1 set (12 randomized points)	2 sets (24 randomized points)	3 sets (36 randomized points)
Passive ROM^a^ training			
Position holding duration	5 seconds	10 seconds	15 seconds
Number of repetitions per set	2×10	3×10	4×10
JPS^b^ training			
Number of repetitions	5	7	9
Active ROM training			
Number of sets	5×7	7×7	9×7

a ROM: range of motion.

b JPS: joint position sense.

#### Stage 2: Passive ROM Training

At the beginning of each session, a brief presession safety calibration will be performed to determine the participant’s session-specific safe and tolerable ROM for dorsiflexion combined with eversion. Passive ROM training will then be delivered within 80% of this session-specific ROM to avoid end-range loading [22].

The default passive movement speed will be set at 6° per second to minimize reflex responses, considering the velocity-dependent nature of spasticity [15]. Before training, 2 familiarization or test cycles will be performed at 6° per second to assess pain, discomfort, increased muscle tone, involuntary contraction, or other signs of intolerance. During these cycles and throughout passive ROM training, real-time sEMG and load-cell signals will be monitored to detect excessive muscle activation, increased tone, abnormal force responses, or uncontrolled movements, thereby supporting therapist supervision and safety. If the default speed is not tolerated, the movement speed may be reduced for that session, and the deviation will be documented.

During passive ROM training, the robot will move the participant’s ankle into dorsiflexion combined with eversion, maintain the end position for a specified duration (position holding duration), and then return the ankle to neutral. During this stage, the participant will track the VR game displayed on the screen. The VR game is based on a standardized reference trajectory for passive ROM training. In the visual scenario, the collection of “gold” and “diamonds” by a bird is temporally synchronized with the ankle motion: the bird moves upward during dorsiflexion and downward during the return to neutral, allowing the participant to match the passive joint movement with immediate visual feedback (Figure 4). Progression will be achieved by gradually increasing the position-holding duration and the number of sets. Each set will comprise 10 passive movement cycles (Table 2).

#### Stage 3: Proprioception or JPS Training

JPS training will be delivered using an ipsilateral joint position paradigm. Prior to each session, session-specific active ankle ROM will be determined, and all target angles will be selected within this safe active range, taking into account spasticity, pain, and joint limitations. For all trials, participants will indicate task completion by pressing a handheld button with the unaffected hand; the ankle position will then be held for approximately 2 seconds to allow acquisition of a stable angular sample before returning to neutral. Target and reproduced or detected angles will be recorded using the robot’s onboard kinematic sensing, and angular error will be computed. Error feedback will be provided in a standardized manner according to the protocol progression (Table 2). The training program will use a graded progression from sensory-focused processing to sensorimotor integration over 6 weeks.

##### Weeks 1 to 3 (Passive Positioning; Sensory-Focused Phase)

During the first 3 weeks, the protocol will exclusively use passive positioning to emphasize processing of external afferent feedback and joint position awareness without the confounding influence of motor deficits (eg, spasticity or weakness). For each trial, the robot will passively move the participant’s paretic ankle toward predefined dorsiflexion target angles within the session-specific active ROM. The participant will press the handheld button as soon as they perceive that the target angle has been reached (passive detection). Visual conditions will be eyes open with VR feedback, and the VR task will provide synchronized visual information by mapping dorsiflexion to the bird’s real-time upward-downward motion (Figure 6), supporting cross-modal calibration of joint position perception.

##### Weeks 4 to 6 (Active Reproduction; Sensorimotor Integration Phase)

During the final 3 weeks, the protocol will transition to an active joint position reproduction task. As in the passive phase, the robot will first passively position the paretic ankle at the target dorsiflexion angle and return it to neutral. The participant will then be instructed to actively reproduce (match) the previously presented dorsiflexion angle using the same (paretic) ankle and press the handheld button when they believe the target has been reached (active reproduction). During this phase, visual feedback will be removed (eyes closed) to promote reliance on proprioceptive afferent input; no visual cues will be provided, and error feedback may be presented at the end of each trial. Although the protocol is planned as eyes open for the first 3 weeks and eyes closed for the final 3 weeks, transition to and continuation of the eyes-closed phase will be determined by the participant’s performance, tolerance, and safety limits.

Progression will include (1) introducing additional target angles within the participant’s session-specific active ROM and (2) using smaller angular differences between target positions when tolerated. However, progression will not rely solely on narrowing angular increments; for some participants, training will initially emphasize improving accuracy at the same target angles before introducing smaller increments.

### Stage 4: Active ROM Training

**Figure 7.**
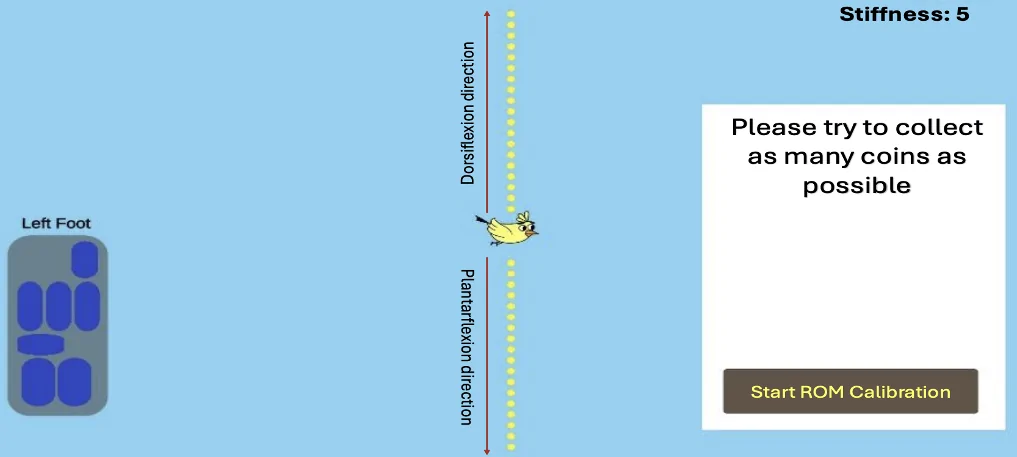
Calibration mode in the virtual reality environment used to set the initial therapy difficulty. ROM: range of motion.

During this phase, repetitive active dorsiflexion-plantarflexion cycles will be performed with the robot adjusting speed and stiffness in real time based on the participant’s performance. At the beginning of each session, a 3-step calibration process will individualize training parameters (Figure 7): (1) determination of a safe ROM under low-speed and low-stiffness settings, with all subsequent active training performed within 80% of this session-specific ROM to avoid end-range loading; (2) gradual stiffness increase within this 80% ROM safety range to identify the highest stiffness level at which the participant can collect all targets during dorsiflexion-plantarflexion cycles; and (3) identification of the maximum movement speed at which the participant can successfully collect all targets over 10 cycles. This calibration establishes a safe and individualized starting point for the session.

Visual feedback will be provided through the synchronized VR task during active movement (Figure 5). The program will emphasize speed training in the initial weeks (motor control focus) and stiffness training in later weeks (strength or stability focus). Transition from speed to stiffness training will be initiated once a performance plateau is reached, defined as <20% change in average speed over the previous 3 weeks. Weekly sets will be progressively increased (Table 2), and each set will consist of 7 repetitions of active dorsiflexion–plantarflexion cycles.

### Stage 5: Sensory Education—Plantar Vibration and Sensory Localization Training

Training will be completed by repeating stage 1.

### Manual Training Protocol

#### Overview

The control intervention will consist of a structured ankle-foot training protocol delivered manually by a physiotherapist. The safety principle will be applied clinically using presession electrogoniometer measurements and the physiotherapist’s judgment in this group. Safety will be maintained through a standardized manual procedure, including consistent participant positioning, slow and controlled movement, electrogoniometer-guided target-angle selection, and continuous monitoring of pain, resistance, tone, and participant tolerance. Target angles will be selected within a comfortable, pain-free range and will avoid end-range loading. The stages and progression of the training protocol are described in the following sections.

#### Stage 1: Sensory Education

Sensory training will consist of 2 parts. In the first part, a plantar massage will be applied to the sole of the foot by a physiotherapist. The massage will be delivered using both thumbs in a rhythmic, pressure-controlled manner without sliding. It will be performed at an intensity that does not cause pain, moving from distal to proximal, using a combination of kneading (petrissage) and stroking (effleurage) techniques. No standardization will be applied regarding the time allocated to petrissage versus effleurage or the duration spent on specific plantar regions; total massage duration will be 3 minutes [43].

In the second part, sensory localization training will be administered. Using a Semmes-Weinstein monofilament, one set will comprise 12 randomized stimulations delivered across the same 7 plantar regions (eg, 1-7-5-3-1-2-4-6-5-7-3‐4). Monofilament applications will be delivered using a standardized contact time and pressure to ensure consistent stimulation. The stimulus duration and the fixed 5-second rest or response period will match the robotic group (Table 2), with responses recorded by the physiotherapist. Progression will be achieved by reducing stimulus duration and increasing the number of sets in accordance with Table 2.

#### Stage 2: Passive ROM Training

During passive ROM training, the physiotherapist will manually move the ankle into dorsiflexion with eversion and hold this position for a specific period (position holding duration). The ankle will then be returned to the neutral position. Progression at this stage will be achieved by gradually increasing the position holding duration at the end of the passive movement and the number of sets (Table 2). Each set will consist of 10 passive movement cycles. The initial movement speed for training will be individually determined for each participant as the speed at which the full ROM can be safely and comfortably completed, as judged by the physiotherapist, and this participant-specific speed is planned to be maintained throughout the protocol. To improve standardization, passive movement speed will be monitored using a timer and kept consistent across sessions within each participant.

#### Stage 3: Proprioception or JPS Training

##### Training Procedures

The sequencing, set-repetition structure, target-angle determination, progression steps, and the eyes-open or eyes-closed schedule will be the same as in the Robotic Education Group. However, target-angle presentation, passive movement of the ankle to the target angles within the session-specific active ROM, return to neutral, and task instruction will be delivered manually by the physiotherapist rather than by the robot.

##### Weeks 1 to 3 (Passive Detection)

For each trial, the physiotherapist will passively move the ankle toward the predefined target angle, and the participant will indicate perceived target attainment verbally (eg, “stop or reached”). The position will then be held for approximately 2 seconds and returned to neutral.

##### Weeks 4 to 6 (Active Reproduction)

After passive presentation of the target angle and return to neutral, the participant will actively reproduce the target angle using the paretic ankle and will indicate perceived target attainment verbally, followed by a ~2-second hold.

Joint angles will be recorded using an electrogoniometer, and angular error will be calculated in accordance with the robotic group procedure. Feedback on error will be provided at the end of each trial.

### Stage 4: Active ROM Training

The patient will be asked to actively perform dorsiflexion and plantarflexion at angles determined by the physiotherapist using an electrogoniometer. Progression in the active ankle training program will consist of speed training in the first weeks to improve motor control, followed by strength or stiffness training in later weeks to increase muscle strength and stability. Progress will be achieved through instructions given to the patient to gradually increase movement speed according to each individual’s tolerance and capacity. Transition to strength training with physiotherapist-applied manual resistance will occur when the physiotherapist observes that the patient can perform the task at higher speeds while maintaining movement quality and can respond effectively to the applied resistance. The number of weekly sets will be gradually increased, with each set planned to consist of 7 repetitions of active dorsiflexion and plantarflexion movements.

### Stage 5: Sensory Education

The training will be completed by repeating the first stage.

### Outcomes

#### Overview

Baseline demographic characteristics (age, sex, stroke date, and affected side) will be collected by self-report, and height and weight will be measured. Clinical and physical outcome measures will be assessed by a physiotherapist blinded to group allocation at baseline (T0) and immediately post-intervention (T1, end of week 6) using standardized assessment procedures.

#### Primary Outcome

The primary outcome will be as follows: *walking speed* will be assessed using the 10MWT. The test will be performed on a 14-m-long, flat, unobstructed walkway including 2 m acceleration and 2 m deceleration distances. Time (seconds) will be recorded between 2 and 12 m using a stopwatch (dynamic start method), and walking speed (m/s) will be calculated as 10 m divided by the recorded time. Participants may use their customary walking aids. The test will be performed 3 times, and the mean walking speed will be used for analysis [44,45].

#### Secondary Outcomes

The secondary outcomes will be as follows.

*Passive and active ankle ROM* will be assessed using an electrogoniometer. Participants will be positioned on an examination plinth with the hip and knee flexed to 90°, tibiae perpendicular to the floor, and feet unsupported. Neutral ankle position will be defined as 0° (foot perpendicular to the tibia). For dorsiflexion-plantarflexion measurements, the axis will be aligned with the lateral malleolus, the proximal reference with the lateral midline of the fibula, and the distal reference with the lateral midline of the fifth metatarsal. The rearfoot will be stabilized to minimize inversion-eversion during testing [46].

*Motor performance* will be assessed using the FMA-LE. The FMA-LE evaluates hip, knee, and ankle motor function across reflex activity, synergies, selective movements, and coordination or speed. Items are scored 0 to 2 (total 0‐34), with higher scores indicating better motor function. Assessments will be conducted in a standardized, quiet environment using standardized verbal instructions in supine, sitting, or standing positions as required by the test [36].

*Walking capacity* will also be assessed using the 2-minute walk test distance (m). Participants will walk for 120 seconds at a self-selected, comfortable, and safe pace. Customary walking aids may be used if needed, but no manual assistance will be provided. Rest breaks will be allowed; however, the timer will continue uninterrupted. The total distance covered will be recorded in meters [47].

*Muscle tone* will be assessed using the Modified Ashworth Scale for the quadriceps, hamstrings, adductors, and gastrosoleus. Modified Ashworth Scale scores range from 0 to 4 (including 1+), with lower scores indicating less spasticity: 0 (no increase in tone), 1 (slight increase in tone), 1+ (marked resistance through less than half of the ROM), 2 (increased tone through most of the ROM), 3 (considerable increase in tone making passive movement difficult), and 4 (rigidity). Assessments will be performed with a single, rapid passive movement through ROM with the participant relaxed [21].

*Ankle JPS* accuracy will be assessed using a standardized protocol with manual application and electrogoniometer-based measurement. Absolute error (AE) will be calculated as AE=|target angle−reproduced angle| and averaged across repeated trials for each target angle; the mean across target angles will be reported as total AE. Assessments will be performed in a seated position with hip and knee flexed at approximately 90°. Visual cues will be eliminated by asking participants to keep their eyes closed or using an eye mask. The electrogoniometer will be fixed using the tibia and dorsum of the foot as reference landmarks, and testing will use dorsiflexion target angles selected within the presession active ROM and from the mid-range of available ROM (eg, 30%‐70% of active ROM) to reduce end-range mechanical cues. The same target angles will be used at all assessment time points. Passive movement speed will be standardized using a stopwatch (2 s to target, 3 s to hold, and 2 s to return to neutral), followed by a 2-second pause in neutral. The assessor will then move the ankle passively again in the same direction, and the participant will indicate when they perceive the target angle by saying stop. The angle at that moment will be recorded, and the AE will be calculated. If velocity-dependent increases in spasticity or fatigue are observed, passive movement speed may be reduced and deviations documented. A minimum of 3 trials (preferably 4) will be performed per target angle, with 15 to 20 seconds of rest between trials and 30 to 60 seconds of rest between target angles. The target-angle order will be randomized and kept consistent across time points. In cases of severely limited active dorsiflexion ROM, a single participant-specific mid-range target angle (eg, 5° DF or another tolerable mid-range value) will be used [7,10,48-50]. Active, performance-based training will be used to promote sensorimotor learning, whereas JPS will be assessed using a passive paradigm to minimize confounding effects of strength, spasticity, and motor control on reproduction accuracy.

*Static balance* will be assessed using the Single-Leg Stance Test. The test will be performed separately for each limb; 3 trials will be recorded per limb and averaged (s). Testing will occur on a flat, nonslip surface in a safe and quiet environment. Timing will start when the foot is fully lifted and stop when balance is lost, the foot touches down, or support is used [51].

*Dynamic balance and functional mobility* will be assessed using the Timed Up and Go test. The time (in seconds) required to stand from a chair, walk 3 m, turn, return, and sit will be recorded. Participants may use their habitual walking aids if needed; no manual assistance will be provided. Three trials will be averaged [52]. The Mini Balance Evaluation Systems Test will assess dynamic balance across anticipatory postural adjustments, reactive postural control, sensory orientation, and dynamic gait. Each of the 14 items is scored 0 to 2 (total 0‐28), with higher scores indicating better balance [53,54].

*Tactile perception* will be assessed using the Semmes-Weinstein Monofilament Test. Five monofilaments will be applied to plantar (7 points) and dorsal (2 points) regions; analyses will use changes in the corresponding force (g) values. Participants will be supine with eyes closed. The monofilament will be applied perpendicular to the skin for ~1‐1.5 seconds; 3 repetitions will be performed at each point and responses recorded verbally [55].

*The Stroke-Specific Quality of Life Scale* will be used to assess QoL. The *Stroke-Specific Quality of Life Scale* is a valid and reliable instrument comprising 12 domains and 49 items, covering the physical, functional, and psychosocial aspects of individuals after stroke. Assessments will be conducted in a quiet environment based on participants’ self-reports, with items scored using a 5-point Likert scale. Total scores range from 49 to 245, with higher scores indicating better QoL. The scale will be administered before and after the intervention, and changes in total and/or subscale scores will be used for analysis [56,57].

### Data Collection and Management

All assessment data will be entered directly into an electronic database (Microsoft Excel) and stored as encrypted files. Data processing and storage will be conducted in accordance with the Turkish Personal Data Protection Law 6698. Electronic data will be kept on password-protected computers and in encrypted folders accessible only to authorized research personnel at Istanbul Medipol University. Paper documents (eg, informed consent forms) will be stored in locked archive cabinets within the Department of Physiotherapy and Rehabilitation. Each participant will be assigned a unique study identification number, and personally identifiable information will be stored separately from research data. To maintain assessor blinding, the outcome assessor will not have access to randomization codes or intervention logs. To promote data quality, the assessor will receive training on standardized operating procedures prior to participant enrollment. Where applicable, outcomes will be based on repeated trials and averaged values (eg, 3 trials for walking and mobility tests with the mean value used for analysis) to improve measurement reliability. The clinical instruments and questionnaires used in this trial are widely applied in stroke rehabilitation and have established reliability and validity in people with stroke (see the relevant references for each measure in the “Outcomes” section). Data accuracy and integrity will be monitored through weekly quality checks, including verification for completeness and plausibility (eg, expected ranges and formats), and electronic data will be regularly backed up.

### Statistical Analysis

Descriptive statistics will be reported as frequency and percentage for categorical variables and as mean (SD) for continuous variables. For nonnormally distributed continuous variables, median and IQR will also be reported. Distributional characteristics will be evaluated using skewness and kurtosis values, together with visual inspection of histograms and Q-Q plots.

The primary analysis will follow the intention-to-treat principle and will include all randomized participants according to their allocated groups. For the primary outcome, 10MWT-derived walking speed, between-group differences at post-intervention will be analyzed using an analysis of covariance (ANCOVA) model, with group as the main factor and baseline walking speed as a covariate. The stratification variables used in randomization, namely baseline FMA-LE severity and affected hemisphere, will also be included as covariates. Model assumptions will be checked before interpretation of the ANCOVA results. Effect estimates will be reported as adjusted mean differences with 95% CIs and effect sizes.

The secondary continuous outcomes will be analyzed similarly using ANCOVA models adjusted for the corresponding baseline value and the same stratification variables. Categorical variables will be compared using chi-square test or Fisher exact test when expected cell counts are small.

Missing outcome data will be described, and reasons for missingness will be documented. If post-intervention primary outcome data are missing, multiple imputation will be used under the missing-at-random assumption for the primary intention-to-treat analysis. A complete-case analysis will also be performed as a sensitivity analysis. In addition, a per-protocol analysis will be conducted as a sensitivity analysis, including participants who complete at least 70% of the intervention sessions, have post-intervention outcome data, and have no major protocol deviations.

The primary outcome will be tested at a 2-sided α level of .05. The secondary outcomes will be considered exploratory and supportive of the primary outcome; therefore, no formal correction for multiple secondary outcomes will be applied. These findings will be interpreted cautiously and reported with effect estimates and 95% CIs. No interim efficacy analyses are planned. Analyses will be performed by an independent statistician according to a prespecified statistical analysis plan.

### Oversight and Monitoring

Oversight will be provided by a coordination team comprising the principal investigator, a physiatrist, and a research coordinator. The physiatrist will confirm eligibility and make safety-related decisions regarding continued participation. Recruitment, protocol adherence, and data completeness will be reviewed weekly, and study progress will be discussed in biweekly meetings. Participant safety and adverse events will be monitored throughout the trial; serious adverse events will be reported to the ethics committee in accordance with institutional procedures. Given the low-risk nature of the intervention, short intervention period, and modest sample size, a formal independent Data Monitoring Committee will not be convened; safety oversight will be conducted by the coordination team as described earlier.

### Adherence

Supportive strategies will be implemented to promote adherence and minimize dropout. Before enrollment, participants will be informed about study procedures and expectations. Session scheduling will be standardized while accommodating participants’ daily routines and fatigue levels. To support attendance, participants will receive appointment reminders via phone calls, emails, and/or text messages, and attendance will be monitored throughout the intervention. Missed sessions will be rescheduled within the same week whenever feasible. Participants who discontinue the intervention will be contacted and invited to complete post-intervention outcome assessments (T1) whenever feasible.

### Concomitant Care

To enable an isolated assessment of intervention effectiveness, participants will not be permitted to receive any additional physical therapy, rehabilitation, or structured exercise programs targeting the lower extremities (hip, knee, ankle, and foot) during the study. This restriction includes manual therapy, therapeutic exercises, electrotherapy, and similar lower-extremity interventions. The restriction applies only to lower-extremity physical interventions; participants may continue treatments for systemic health conditions, speech therapy, neuropsychological interventions, and routine medical follow-ups. The use of additional robotic devices or experimental treatments that could directly or indirectly affect lower-extremity function is prohibited. If an unforeseen need for additional lower-extremity treatment arises, it will be reported to the principal investigator and managed according to the study protocol.

### Criteria for Discontinuing or Modifying the Study

Participants may be withdrawn, or the assigned intervention may be discontinued or modified, in the following situations: (1) a serious adverse event or clinically significant complication (eg, cardiovascular instability, sudden neurological deterioration, syncope, medical emergency, or acute pain increase); (2) intolerable pain, muscle spasms, or excessive fatigue; or (3) unexpected clinical changes preventing continued participation. Decisions will be made by the principal investigator in consultation with the physiatrist, prioritizing participant safety and clinical status. Participants may withdraw at any time without providing a reason. Data collected up to withdrawal will be stored in encoded form in accordance with confidentiality principles.

### Harms

All AEs potentially related to the intervention, or expected in this population, will be systematically monitored and documented. AEs will be assessed through (1) active inquiry about new or worsening symptoms at the start of each session, (2) direct observation of discomfort or physical strain during sessions, and (3) participant self-report at any time (telephone, text, or email). Anticipated AEs include musculoskeletal pain, joint stiffness, skin irritation from device contact, overexertion, fatigue, emotional frustration, and transient increases in muscle tone or spasticity. AEs will be recorded by the treating physiotherapist at each session using a standardized AE log; events between sessions will be captured through participant contact and, when needed, review of relevant clinical records. Each AE will be documented (onset, duration, severity, action taken, outcome, and relatedness). Serious adverse events will be reported immediately to the principal investigator and, where required, to the ethics committee, and all AEs will be classified and reported in study publications.

### Ethical Considerations

#### Research Ethics Approval

The initial study protocol, informed consent forms, and study-related documents were approved by the Istanbul Medipol University Clinical Research Ethics Committee (approval E-66291034‐202.3.02-8175) on December 4, 2025. The current approved protocol is version 4, dated January 2026, and was approved by the same ethics committee on January 14, 2026. Written informed consent will be obtained from all participants before enrollment. The consent process will be conducted by physiotherapists trained in research ethics and legal requirements; participants will have the opportunity to ask questions before signing. The study is registered at ClinicalTrials.gov (identifier: NCT07091045). Study findings will be disseminated through peer-reviewed publications and scientific conference presentations.

#### Protocol Amendments

Any protocol modifications that may affect participant safety, burden, or expected benefits will be submitted to the ethics committee for approval prior to implementation. Participants will be informed of relevant changes, and reconsent will be obtained when necessary. All amendments will be documented using updated protocol version numbers and dates and reported in accordance with applicable regulatory requirements.

#### Confidentiality and Access to Data

Access to identifiable medical records will be restricted to authorized personnel at Istanbul Medipol University and Medipol Health Group Hospitals when required for study verification and regulatory compliance. All personnel with access to such records will be bound by professional confidentiality obligations. Participant data will be handled in accordance with applicable data protection legislation (including the Personal Data Protection Law) and relevant ethical standards. Study data will be coded, and identifying information will be stored separately from research datasets. Data collection will be limited to the clinical assessments and outcome measures defined in the protocol. Analyses will be conducted on deidentified datasets. Data will be used solely for the purposes of this research. No identifiable participant data will be shared publicly.

#### Dissemination Policy

Study results will be disseminated regardless of direction or statistical significance via international peer-reviewed publications, presentations at neurological rehabilitation conferences, and inclusion in a PhD dissertation. Individual participant data will be presented only in anonymized form using study codes. A summary of overall study findings may be provided to participants upon request in a clear, nontechnical format. Data analysis and reporting will be completed following study completion and disseminated through appropriate scientific channels. Any educational materials or professional training outputs developed from the findings will be based on aggregated, anonymized results.

## Results

This study is supported by the Scientific and Technological Research Council of Turkey (TÜBİTAK) under the 1002-A Rapid Support Program (grant 225S390). The study protocol was conditionally approved for funding on August 22, 2025, and final funding approval was granted following revisions on January 16, 2026. Recruitment began in March 2026 and is planned to continue until May 2027. As of March 25, 2026, four participants have been enrolled and are currently receiving the assigned intervention. Data analysis will begin once all enrolled participants have completed post-intervention assessments.

## Discussion

This study protocol describes an assessor-blinded, parallel-group randomized controlled trial designed to evaluate the effectiveness of a structured robot-assisted ankle-foot sensorimotor training program compared with a content-matched manual training program in individuals with chronic stroke. The proposed intervention addresses persistent lower-extremity motor and somatosensory impairments after stroke by integrating passive ROM or stretching, plantar vibrotactile localization training, VR-based proprioceptive training, and active or active-assisted ankle ROM training within a single standardized program. By targeting both sensory and motor components of ankle-foot dysfunction, this trial aims to provide clinically relevant evidence for a distal, ankle-foot–focused robotic rehabilitation approach.

A key rationale for the present protocol is that post-stroke gait and balance impairments are not solely motor problems but also reflect altered somatosensory input, impaired proprioception, abnormal muscle tone, weakness, and reduced motor control [7,10,55,56]. The plantar surface and ankle provide important sensory information for postural orientation, weight transfer, and rapid postural corrections [7,27,49]. Therefore, rehabilitation strategies that address ankle mobility and motor control together with plantar tactile and proprioceptive processing may be particularly relevant for improving functional walking and balance [10,49,50]. In this context, the BalanSENS protocol differs from many previous ankle robotic interventions by embedding plantar vibrotactile localization and proprioceptive training into the same robot-assisted rehabilitation framework rather than applying sensory stimulation as an isolated modality.

Another important feature of the protocol is the structured integration of passive and active training components. Passive ROM and stretching are commonly used to maintain or improve ankle mobility and reduce resistance to movement, but passive repetition alone may be insufficient to optimize motor learning [21,58]. Therefore, the proposed program progresses from passive and sensory-focused elements toward active, performance-based ankle training. During active ROM training, VR-based tasks and real-time performance-sensitive adaptation are used to promote active engagement and individualized task difficulty. The assist-as-needed strategy is expected to support participation by providing assistance when required while encouraging voluntary motor output. This approach is consistent with contemporary motor learning principles emphasizing task specificity, sufficient repetition, active engagement, feedback, and individualized progression [58].

A further strength of this trial is the use of a manual training group that is designed to be content matched to the robotic intervention. Many studies compare robotic rehabilitation with usual care or broadly defined conventional therapy, which may make it difficult to interpret whether observed effects are related to robotic delivery itself or to differences in dose, structure, attention, or task content [35,40]. This design may provide a more direct evaluation of the added value of robot-assisted delivery, including standardized trajectory control, VR-based feedback, real-time adaptation, and sensor-supported safety monitoring.

In this study, training dose, session structure, progression rules, ROM safety limits, and feedback conditions are predefined. The use of a standardized robot-assisted protocol may also help address the heterogeneity reported in previous ankle rehabilitation studies. In the robot-assisted group, session-specific calibration will allow training parameters to be individualized while maintaining standardized safety boundaries. This balance between standardization and individualization may improve both reproducibility and clinical applicability. In summary, the findings of this trial may contribute to the development of more standardized, individualized, and sensorimotor-oriented rehabilitation protocols for improving gait and balance after stroke.

## Supplementary material

10.2196/104766 Checklist 1 SPIRIT checklist.

10.2196/104766 Peer Review Report 1 Peer review report from the Scientific and Technological Research Council of Turkey (TÜBİTAK; project 225S390).

## Data Availability

Data used in this study may be obtained from the corresponding author upon reasonable request.

## Abbreviations

10MWT: 10-meter walk test
AAN: assist-as-needed
AE: adverse event
ANCOVA: analysis of covariance
CONSORT: Consolidated Standards of Reporting Trials
FMA-LE: Fugl-Meyer Assessment–Lower Extremity
JPS: joint position sense
MTG: manual training group
QoL: quality of life
ROM: range of motion
RTG: robot-assisted training group
sEMG: surface electromyography
SPIRIT: Standard Protocol Items: Recommendations for Interventional Trials
VR: virtual reality

## References

[1] Sacco RL, Kasner SE, Broderick JP (2013). An updated definition of stroke for the 21st century: a statement for healthcare professionals from the American Heart Association/American Stroke Association. Stroke.

[2] Feigin VL, Stark BA, Johnson CO (2021). Global, regional, and national burden of stroke and its risk factors, 1990–2019: a systematic analysis for the Global Burden of Disease Study 2019. Lancet Neurol.

[3] Arene N, Hidler J (2009). Understanding motor impairment in the paretic lower limb after a stroke: a review of the literature. Top Stroke Rehabil.

[4] Li S, Francisco GE, Zhou P (2018). Post-stroke hemiplegic gait: new perspective and insights. Front Physiol.

[5] Li S (2020). Ankle and foot spasticity patterns in chronic stroke survivors with abnormal gait. Toxins (Basel).

[6] Mahmoudzadeh A, Nakhostin Ansari N, Naghdi S (2020). Effect of ankle plantar flexor spasticity level on balance in patients with stroke: protocol for a cross-sectional study. JMIR Res Protoc.

[7] Cho JE, Kim H (2021). Ankle proprioception deficit is the strongest factor predicting balance impairment in patients with chronic stroke. Arch Rehabil Res Clin Transl.

[8] Xu J, Witchalls J, Preston E (2025). Ankle joint position sense acuity differences among stroke survivors at three walking ability levels: a cross-sectional study. Front Neurol.

[9] Parsons SL, Mansfield A, Inness EL, Patterson KK (2016). The relationship of plantar cutaneous sensation and standing balance post-stroke. Top Stroke Rehabil.

[10] Johnson CA, Biswas P, Tapia R (2025). The weak relationship between ankle proprioception and gait speed after stroke: a robotic assessment study. Neurorehabil Neural Repair.

[11] Forghany S, Nester CJ, Tyson SF (2019). Plantar pressure distribution in people with stroke and association with functional mobility. JRSR.

[12] Covaciu F, Pisla A, Iordan AE (2021). Development of a virtual reality simulator for an intelligent robotic system used in ankle rehabilitation. Sensors (Basel).

[13] Payedimarri AB, Ratti M, Rescinito R, Vanhaecht K, Panella M (2022). Effectiveness of platform-based robot-assisted rehabilitation for musculoskeletal or neurologic injuries: a systematic review. Bioengineering (Basel).

[14] Wang H, Shen H, Han Y, Zhou W, Wang J (2025). Effect of robot-assisted training for lower limb rehabilitation on lower limb function in stroke patients: a systematic review and meta-analysis. Front Hum Neurosci.

[15] Asín-Prieto G, Mercante S, Rojas R (2022). Post-stroke rehabilitation of the ankle joint with a low cost monoarticular ankle robotic exoskeleton: Preliminary results. Front Bioeng Biotechnol.

[16] de la Iglesia DH, Mendes AS, González GV, Jiménez-Bravo DM, de Paz Santana JF (2020). Connected elbow exoskeleton system for rehabilitation training based on virtual reality and context-aware. Sensors (Basel).

[17] Olimb Hillkirk A, Skavberg Roaldsen K, Johnsen HM (2025). Physiotherapists’ user acceptance of a lower limb robotic exoskeleton in specialized rehabilitation: qualitative exploratory study. JMIR Rehabil Assist Technol.

[18] Lee MH, Tian MY, Kim MK (2024). The effectiveness of overground robot exoskeleton gait training on gait outcomes, balance, and motor function in patients with stroke: a systematic review and meta-analysis of randomized controlled trials. Brain Sci.

[19] Alvarez-Perez MG, Garcia-Murillo MA, Cervantes-Sánchez JJ (2020). Robot-assisted ankle rehabilitation: a review. Disabil Rehabil Assist Technol.

[20] Mao Y, Gao Z, Yang H, Song C (2022). Influence of proprioceptive training based on ankle-foot robot on improving lower limbs function in patients after a stroke. Front Neurorobot.

[21] Zhai X, Wu Q, Li X (2021). Effects of robot-aided rehabilitation on the ankle joint properties and balance function in stroke survivors: a randomized controlled trial. Front Neurol.

[22] Cho JE, Lee WH, Shin JH, Kim H (2021). Effects of bi-axial ankle strengthening on muscle co-contraction during gait in chronic stroke patients: a randomized controlled pilot study. Gait Posture.

[23] Varas-Diaz G, Cordo P, Dusane S, Bhatt T (2022). Effect of robotic-assisted ankle training on gait in stroke participants: a case series study. Physiother Theory Pract.

[24] Ferry B, Compagnat M, Yonneau J (2022). Awakening the control of the ankle dorsiflexors in the post-stroke hemiplegic subject to improve walking activity and social participation: the WAKE (Walking Ankle isoKinetic Exercise) randomised, controlled trial. Trials.

[25] Yu Y, Huang W, Tuerxun H (2025). Enhanced neuroplasticity and gait recovery in stroke patients: a comparative analysis of active and passive robotic training modes. BMC Neurol.

[26] Jharbade M, Ramachandran S, V S, Solomon M J (2024). Functional training for lower extremities in stroke survivors: a scoping review. Cureus.

[27] Karimi-AhmadAbadi A, Naghdi S, Ansari NN, Fakhari Z, Khalifeloo M (2018). A clinical single blind study to investigate the immediate effects of plantar vibration on balance in patients after stroke. J Bodyw Mov Ther.

[28] Sajedifar M, Fakhari Z, Naghdi S, Nakhostin Ansari N, Honarpisheh R, Nakhostin-Ansari A (2023). Comparison of the immediate effects of plantar vibration of both feet with the plantar vibration of the affected foot on balance in patients with stroke: preliminary findings. J Bodyw Mov Ther.

[29] Bonassi G, Biggio M, Bisio A, Ruggeri P, Bove M, Avanzino L (2017). Provision of somatosensory inputs during motor imagery enhances learning-induced plasticity in human motor cortex. Sci Rep.

[30] Frank SM, Otto A, Volberg G, Tse PU, Watanabe T, Greenlee MW (2022). Transfer of tactile learning from trained to untrained body parts supported by cortical coactivation in primary somatosensory cortex. J Neurosci.

[31] Park JH (2018). The effects of plantar perception training on balance and falls efficacy of the elderly with a history of falls: a single-blind, randomized controlled trial. Arch Gerontol Geriatr.

[32] Matsuno S, Yoshiike T, Yoshimura A (2021). Contribution of somatosensory and parietal association areas in improving standing postural stability through standing plantar perception training in community-dwelling older adults. J Aging Phys Act.

[33] Suganuma J, Ikeda Y, Chidori K (2024). Effects of foot somatosensory training on plantar somatosensory function. Cureus.

[34] Rinderknecht MD, Dueñas JA, Held JP (2019). Automated and quantitative assessment of tactile mislocalization after stroke. Front Neurol.

[35] Mehrholz J, Pohl M, Platz T, Kugler J, Elsner B (2015). Electromechanical and robot-assisted arm training for improving activities of daily living, arm function, and arm muscle strength after stroke. Cochrane Database Syst Rev.

[36] Kwong PWH, Ng SSM (2019). Cutoff score of the lower-extremity motor subscale of Fugl-Meyer assessment in chronic stroke survivors: a cross-sectional study. Arch Phys Med Rehabil.

[37] Drogos JM, Carmona C, Arceo R, Yao J (2025). Effects of device-assisted practice of activities of daily living in a close-to-normal pattern on upper extremity motor recovery in individuals with moderate to severe stroke: study protocol of a randomized control trial. Trials.

[38] Ersoy T, Hocaoglu E, Kaya P, Unal R (2024). Design of BalanSENS: functional evaluation in ankle preparation phase. J Bionic Eng.

[39] Hocaoglu E, Beyaz M, Ahmed MAT, Gurluk A, Unal R, Kaya P SEMG-based assessment of engagement in VR-integrated balansens ankle rehabilitation.

[40] Forrester LW, Roy A, Krywonis A, Kehs G, Krebs HI, Macko RF (2014). Modular ankle robotics training in early subacute stroke: a randomized controlled pilot study. Neurorehabil Neural Repair.

[41] Horstman A, Gerrits K, Beltman M, Janssen T, Konijnenbelt M, de Haan A (2009). Muscle function of knee extensors and flexors after stroke is selectively impaired at shorter muscle lengths. J Rehabil Med.

[42] Önal B, Sertel M, Karaca G (2022). Effect of plantar vibration on static and dynamic balance in stroke patients: a randomised controlled study. Physiotherapy.

[43] Wikstrom EA, Song K, Lea A, Brown N (2017). Comparative effectiveness of plantar-massage techniques on postural control in those with chronic ankle instability. J Athl Train.

[44] Yeung LF, Lau CCY, Lai CWK, Soo YOY, Chan ML, Tong RKY (2021). Effects of wearable ankle robotics for stair and over-ground training on sub-acute stroke: a randomized controlled trial. J Neuroeng Rehabil.

[45] Bowden MG, Balasubramanian CK, Behrman AL, Kautz SA (2008). Validation of a speed-based classification system using quantitative measures of walking performance poststroke. Neurorehabil Neural Repair.

[46] Johnson CA, Farrens AJ, Biswas P (2025). Robotic ankle assessment post-stroke: reliability, comparison to therapists, and benchmark dataset development. Sensors (Basel).

[47] Cristina da Silva L, Danielli Coelho de Moraes Faria C, da Cruz Peniche P, Ayessa Ferreira de Brito S, Tavares Aguiar L (2024). Validity of the two-minute walk test to assess exercise capacity and estimate cardiorespiratory fitness in individuals after stroke: a cross-sectional study. Top Stroke Rehabil.

[48] Smith TO, Davies L, Hing CB (2013). A systematic review to determine the reliability of knee joint position sense assessment measures. Knee.

[49] Yalcin E, Akyuz M, Onder B, Kurtaran A, Buyukvural S, Ozbudak Demir S (2012). Position sense of the hemiparetic and non-hemiparetic ankle after stroke: is the non-hemiparetic ankle also affected?. Eur Neurol.

[50] Huang Q, Elangovan N, Zhang M, Van de Winckel A, Konczak J (2024). Robot-aided assessment and associated brain lesions of impaired ankle proprioception in chronic stroke. J Neuroeng Rehabil.

[51] Flansbjer UB, Blom J, Brogårdh C (2012). The reproducibility of Berg Balance Scale and the Single-leg Stance in chronic stroke and the relationship between the two tests. PM R.

[52] Ng SS, Hui-Chan CW (2005). The timed up & go test: its reliability and association with lower-limb impairments and locomotor capacities in people with chronic stroke. Arch Phys Med Rehabil.

[53] Tsang CSL, Liao LR, Chung RCK, Pang MYC (2013). Psychometric properties of the Mini-Balance Evaluation Systems Test (Mini-BESTest) in community-dwelling individuals with chronic stroke. Phys Ther.

[54] Göktaş A, Çolak FD, Kar İ, Ekici G (2020). Reliability and validity of the Turkish version of the Mini-BESTest Balance Scale in patients with stroke. tnd.

[55] Sajedifar M, Fakhari Z, Naghdi S, Nakhostin Ansari N, Honarpisheh R (2020). The short term-effects of both feet plantar vibration in post stroke patients balance. AVR.

[56] Williams LS, Weinberger M, Harris LE, Clark DO, Biller J (1999). Development of a stroke-specific quality of life scale. Stroke.

[57] Hakverdioğlu Yönt G, Khorshid L (2012). Turkish version of the Stroke-Specific Quality of Life Scale. Int Nurs Rev.

[58] Coremans M, Allewijn I, Bataillie F (2026). Impact of a personalized, high-dose, intensive motor rehabilitation program, Integrating Advanced Technology for Adults With Central Neurological Conditions (INTeRAcT): protocol for a single-blind randomized controlled trial with a clinical, health economic, and process evaluation. JMIR Res Protoc.

