# Supplementary material for: Effectiveness of a Structured Robot-Assisted Ankle-Foot Sensorimotor Training Program Versus Manual Training in Chronic Stroke: Protocol for an Assessor-Blinded, Parallel-Group Randomized Controlled Trial
Source: JMIR Res Protoc. 2026 Sep 25;15:e104766. doi: 10.2196/104766 (PMC13614211; doi:10.2196/104766)
Supplement: Supplementary file 2 [file resprot-v15-e104766-s002.pdf]

REPUBLIC OF TÜRKİYE
THE SCIENTIFIC AND TECHNOLOGICAL RESEARCH COUNCIL OF TÜRKİYE (TÜBİTAK)
Directorate of Research Support Programs

Dear Esra TEKECİ,

The scientific evaluation process regarding your project numbered **225S390**, titled *"Effectiveness of Robot-Assisted Structured Foot–Ankle Sensorimotor Training in Stroke Patients"*, submitted to our Institution within the scope of the **"1002-A Rapid Support Module"**, has been completed.

As a result of the evaluations conducted by expert referees in the field; it will be possible for your project to be supported **provided that** the revisions related to the issues communicated in the scientific evaluation report are made and that the revisions made are deemed appropriate by our Group.

In order to initiate the contracting process of your project; the project must be revised in line with the issues specified in the scientific evaluation report sent to you, and the revised project proposal (**changes must be indicated in bold**) together with a separate information document explaining the changes made must be uploaded to **"pys.tubitak.gov.tr"** within **15 days** from the date this letter is delivered to you.

If the required revisions are not made within the specified period or if the revisions made are not found sufficient by our Group, the contracting procedures of your project will not be initiated and your project will not be put into effect.

Following the fulfillment of the aforementioned conditions; evaluation studies on financial and similar matters will be initiated within the framework of the relevant legislation. In order for your project contract to be executed, the required documents must be submitted to the relevant Group via the **Panel Management System (PYS)**. After the submission of the documents, the contracts and other related documents will be sent to you for signature.

Respectfully submitted for your information.

*During a person's career, they may serve as a project coordinator a maximum of five times in total within the scope of the 1002-A Rapid Support Module and the 1002-B Emergency Support Module.

Number: B.14.2.TBT.0.06.03.02-161-699972
Date: 22/08/2025
Subject: Decision Letter for Project No. 225S390

Dr. Hatice Mahur TURAN
Health Sciences Research Support Group (SBAG)
Acting Group Coordinator

REPUBLIC OF TÜRKİYE
THE SCIENTIFIC AND TECHNOLOGICAL RESEARCH COUNCIL OF TÜRKİYE (TÜBİTAK)
To the Directorate of Research Support Programs

Our project titled *"Effectiveness of Robot-Assisted Structured Foot–Ankle Sensorimotor Training in Stroke Patients"* with project number **225S390**, submitted within the scope of the **"1002-A Rapid Support Module"** in the first term of 2025, has been re-evaluated in line with the valuable opinions and suggestions of expert reviewers. As a result of these evaluations, the project methodology has been scientifically strengthened, and various revisions have been made to enhance its applicability. Below, the main revisions made based on reviewer comments and their justifications are presented in detail.

INFORMATION DOCUMENT EXPLAINING THE REVISIONS MADE

ORIGINALITY

Scientific evaluation report – Reviewer Comment 1:

In the project proposal, it is planned to examine the effects of robot-assisted foot–ankle sensorimotor training protocol on pain, sensory integration, and motor skills in stroke patients. While robotic applications in foot–ankle rehabilitation in the current literature mostly focus on motor gains, the project proposal has an innovative characteristic by addressing sensory, motor, and cognitive integration within the same protocol. However, how the distinction between sensorimotor training and motor training is defined and how it will be presented in the project proposal has not been sufficiently discussed in light of the literature. This situation has been evaluated as partially limiting the originality of the project.

Revision made:

Taking the expert opinion into consideration, necessary revisions were made to more clearly present the distinction between sensorimotor training and motor training in the project proposal.

In the project text:

In the section **ORIGINALITY, 1.1.1. Importance of the Topic, iii. Development of Lower Extremity Robots in Stroke**, the following statement has been added:

"While motor training after stroke is a fundamental intervention approach that mainly focuses on strength, range of motion, coordination, and repetitive motor performance, sensorimotor training includes the active use and integration of sensory inputs (proprioception, tactile, vestibular) in addition to motor skills. Thus, it aims to increase individuals' level of independence in activities of daily living and improve their quality of life (Hobbs & Artemiadis (2020); Payedimarri et al. (2022); Banyai & Brisan (2024)). While robotic foot–ankle rehabilitation studies in the literature generally focus on motor gains such as walking speed, step length, or muscle strength, sensorimotor approaches have mostly been limited to low-technology methods such as balance

boards, vibrating surfaces, or virtual reality-based proprioceptive exercises (Hoh & Semrau (2025)).”

Additionally, in section **1.1.2. Original Value of the Project**, the following emphasis has been added:

“This project aims to integrate proprioceptive and tactile sensory stimuli into motor control processes rather than focusing solely on motor training. While foot–ankle robotic rehabilitation in the literature generally focuses on motor skills (Hobbs & Artemiadis (2020); Hoh & Semrau (2025)), this study prioritizes sensorimotor integration and addresses motor and sensory components holistically within a structured protocol. Thus, by evaluating motor performance and sensory feedback together through robot-assisted rehabilitation, it will provide an original contribution to the literature.”

METHOD

Scientific evaluation report – Reviewer Comment 2:

Exclusion criteria were not sufficiently detailed.

Revision made:

Exclusion criteria were revised and detailed as follows:

“Exclusion criteria: Cognitive impairment (Mini-Mental State Examination score ≤ 24), presence of pathologies affecting walking or balance (orthopedic complications, lower extremity amputation, osteoporosis, etc.) (Cho et al. (2021)), insufficient visual acuity to see a screen (e.g., diplopia) (Cho et al. (2021)), presence of severe visual problems such as hemianopia and vestibular disorders (Sajedifar et al. (2023)), sensory deficits defined as hemisensory neglect (Forrester et al. (2011)), acute musculoskeletal or cardiovascular disorders (Varas-Diaz et al. (2021)), having received Baclofen pump or Botox treatment within the last 5 months (Varas-Diaz et al. (2021)), fixed or painful contractures in the paretic ankle (Forrester et al. (2011); Khalifeloo et al. (2018); Ferry et al. (2022); Zhang et al. (2023); Majeed et al. (2024)), uncontrolled systemic diseases (e.g., diabetes, hypertension, debilitating or immunosuppressive diseases) (Asín-Prieto et al. (2022)), history of neurological disease other than stroke or having a disorder preventing understanding of instructions (Zhang et al. (2023)). Additionally, individuals receiving concurrent lower extremity physical therapy, robotic rehabilitation, or neurological exercise programs will not be included in the study.”

Scientific evaluation report – Reviewer Comment 3:

The reference used for joint range of motion measurement does not match the method.

Revision made:

The reference has been revised as follows:

“Jung I., Yu I., Kim S., Lee D., Oh J. (2015). Reliability of ankle dorsiflexion passive range of motion measurements obtained using a hand-held goniometer and Biodex dynamometer in stroke patients. J. Phys. Ther. Sci. 27: 1899–1901.”

Scientific evaluation report – Reviewer Comment 4:

The rationale for selecting an effect size of d = 1.135 in the power analysis was not provided.

Revision made:

Based on the expert reviewer’s opinion, the justification for the effect size was added:

“…Based on a reference study (Cho et al., 2021), comparing the mean and standard deviation values of the Fugl-Meyer lower extremity scores between the robot-assisted rehabilitation group (25.8 (4.5)) and the conventional training group (21.5 (2.9)), Cohen’s d was calculated as 1.123. Based on this effect size, a power analysis conducted using G*Power 3.1 determined that a total sample size of n=30 (15 per group) would provide 90% power (α=0.05).”

Scientific evaluation report – Reviewer Comment 5:

The primary outcome measure has not been defined.

Revision made:

Taking the expert reviewer’s evaluation into consideration, the necessary updates have been made in Section 2.1. Materials and Methods of the project proposal. Within this scope, the primary outcome measures of the study have been clearly defined in the project.

Scientific evaluation report – Reviewer Comment 6:

The method of randomization (sealed envelope, computer, etc.) and the stratification variables (tear type, trauma history, degree of retraction, etc.) have not been specified.

Revision made:

Taking the expert reviewer’s opinion into consideration, the method of randomization and stratification has been explained with support from the literature. The following statement has been added to Section 2.1. Materials and Methods of the project proposal:

“In order to ensure baseline balance between groups, stratified permuted block randomization will be applied considering Fugl-Meyer Lower Extremity (0–20 = severe, 21–27 = moderate, 28–34 = mild/good) levels (Kwong et al. (2019); Balasubramanian

et al. (2016)) and the affected hemisphere (right/left). For each stratum, fixed block allocation lists with a 1:1 ratio (block size = 4; final block = 2 when necessary) will be generated in advance, and the allocation sequence will be concealed through a centralized computer-based system (studyrandomizer.com). In the primary analyses, baseline FMA-LE scores will be included in the statistical model as a covariate."

Scientific evaluation report – Reviewer Comment 7:

It has been stated that the study will be single-blind; however, it has not been explained who the blinded evaluator(s) are and how blinding will be maintained.

Revision made:

Taking the expert reviewer's evaluation into consideration, the details of blinding in the project have been clarified. The following statement has been added to Section 2.1. Materials and Methods:

"The study is designed as single-blind; only the clinical evaluator will be blinded. Assessments will be performed solely by an experienced physiotherapist who is unaware of group allocations. Participants and the therapist administering the intervention will not be blinded due to the nature of the intervention. Ensuring that the evaluator remains unaware of group assignments is planned to maintain the objectivity of measurements and to minimize the risk of bias."

Scientific evaluation report – Reviewer Comment 8:

It has not been explained how progression will be ensured in the treatment program. It has not been specified whether participants will continue physiotherapy for stroke. The session contents, duration-dosage scheme, and practitioner qualification criteria of the REG and KEG protocols have not been defined with support from the literature.

Revision made:

Taking the expert reviewer's evaluation into consideration, the session contents, duration, dosage, and practitioner qualification criteria of the training protocols have been detailed with support from the literature; additionally, how progression will be ensured in the treatment program has been clarified.

In Section 2.1. Materials and Methods of the project, under the heading Characteristics of Training Protocols, the following statement has been revised as:

"Within the scope of this study, all participants will attend conventional rehabilitation sessions lasting approximately 20–25 minutes, three times per week for a total of six weeks. The conventional program will include upper extremity mobilization, flexibility and positioning exercises for spasticity, as well as balance, weight-shifting, and gait training (Ferry et al. (2022)). In addition, while the intervention group (REG) will receive robot-assisted foot–ankle training, the control group (MEG) will participate in manual foot–ankle training administered by a physiotherapist. The duration of these trainings will be approximately 30–35 minutes, and the total session duration will be equalized to 50–60 minutes for both groups. In the literature, robot-assisted interventions conducted three times per week for six weeks with durations of 50–60 minutes have been reported to provide significant improvements in gait biomechanics, balance, joint

position sense, and lower extremity motor functions (Forrester et al., 2016; Asín-Prieto et al., 2022; Ferry et al., 2022). All interventions will be administered by physiotherapists with at least two years of experience in clinical neurorehabilitation and who are competent in the use of robotic systems (Mehrholz et al. (2018)).”

In addition, since the concept of conventional physiotherapy is used here, the control group previously referred to as the “conventional training group (KEG)” in the project text has been renamed as the “manual training group (MEG),” in which the manual foot–ankle training protocol is applied, and it has been indicated accordingly in the relevant sections of the project text.

In Section 2.1. Materials and Methods of the project, the progression process of the training protocols for both groups has been explained in detail with support from the literature.

Scientific evaluation report – Reviewer Comment 9:

It has not been sufficiently explained at which time points the outcome measurements (pain, motor skills, sensory integration, monofilament test, etc.) will be performed, how they will be conducted, and the follow-up timeline.

Revision made:

Taking the expert reviewer’s evaluation into consideration, the explanations regarding outcome measurements have been revised. In the project, the following statement has been added to the **Clinical Evaluation Methods** section under **2.1. Materials and Methods**:

“Clinical and physical outcome measures will be evaluated by a physiotherapist before the intervention (in the 1st week) and after the intervention (at the end of the 6th week).”

Scientific evaluation report – Reviewer Comment 10:

It has been stated that the treatment stages performed with the robot will be evaluated using EMG signals. However, it has been considered a significant limitation that no information has been provided regarding what preprocessing steps such a highly noise-prone biopotential signal like EMG will undergo and which signal processing methods will be used to influence decision-making/support mechanisms.

Revision made:

Taking the expert reviewer’s opinion into consideration, detailed information regarding the preprocessing steps and signal processing methods of EMG biopotential signals has been added to the project text. The relevant text has been indicated in bold in the **secondary outcome measures – tone assessment** section of **2.1. Materials and Methods**:

“In this project, surface electromyography (sEMG) signals will be recorded in real time using Biosignal Plux© EMG sensors. Electrodes will be placed on the tibialis anterior muscle (dorsiflexion) and gastrocnemius muscle (plantar flexion), which are responsible for controlling ankle joint movements. Signals will be collected at a sampling frequency of 2000 Hz and transferred to the MATLAB environment using the

Lab Streaming Layer (LSL) communication protocol. Within the scope of signal preprocessing, a fourth-order Butterworth band-pass filter (20–500 Hz) will be applied to eliminate motion artifacts and high-frequency noise components. Subsequently, full-wave rectification will be performed to obtain the absolute amplitude of muscle activity. In order to reduce the effect of transient amplitude deviations caused by sudden muscle contractions in the rectified signal, the conditional mean of the signal will be calculated using a real-time moving window method. In the final stage, envelope extraction using a low-pass filter will be applied to obtain the amplitude–time profile of muscle activation and to provide a reliable assessment of the patient's contraction dynamics. Additionally, in order to minimize differences between individuals and sessions and to enable accurate comparisons between therapies, the obtained sEMG amplitude values will be normalized according to a reference muscle activation (e.g., maximum voluntary contraction, MVC)."

PROJECT MANAGEMENT

a. Management Structure:

Scientific evaluation report – Reviewer Comment 11:

Although the work packages and their responsible persons appear appropriate, the contribution percentages of the success criteria within the work packages are not clearly defined. The time allocated to Work Package 1 has been considered short considering its place in the project structure. It has been determined that the second evaluation of the first participant in Work Package 5 could begin in the 10th month. Therefore, the time definition of this work package has been evaluated as inappropriate. The project team is considered competent to successfully complete the project.

Revision made:

In line with the expert reviewer's evaluation, contribution percentages have been added to the work packages. Changes have been made to the project timeline. The contributions of the work packages to the success of the project have been determined as 15% for Work Packages 1, 2, 5, and 6, and 20% for Work Packages 3 and 4. While the time intervals of Work Packages 1 and 2 have been extended, the time interval of Work Package 5 has been shortened, and the time interval of Work Package 6 has also been revised. The changes made have been indicated in bold in the work schedule under **2.1. Management Structure: Work Packages (WP), Task Distribution, and Durations**.

b. Risk Management:

Scientific evaluation report – Reviewer Comment 12:

There are inconsistencies between risks and work package numbers. In the case of not reaching a sufficient number of patients, from which centers patients will be recruited remains unclear. Risks related to equipment failure have not been found appropriate.

Revision made:

In line with the expert reviewer’s evaluation, work package numbers have been revised. Centers from which patients will be recruited in case of not reaching a sufficient number of patients have been added, thus eliminating uncertainty. Risks related to equipment failure that were not considered appropriate by expert reviewers have been removed from the project. The changes made have been indicated in bold in the Risk Management section and in the risk management table.
